# Plasma Ephrin-A1 level in a cohort of diabetic retinopathy patients

**DOI:** 10.1186/s12886-020-01580-0

**Published:** 2020-08-05

**Authors:** Danna Mao, Ying Hu, Qi Bao, Kewei Wu, Yaoding Zheng, Yukun Yang, Bo Lei, Ying Jiang

**Affiliations:** 1 Department of Ophthalmology, Medical community of Fenghua Hospital of Traditional Chinese Medicine, 22 Zhong Shan Road, Feng Hua District, NingBo, 315500 ZheJiang China; 2 Department of Clinical Laboratory, Medical community of Fenghua Hospital of Traditional Chinese Medicine, Ningbo, Zhejiang China; 3 People’s Hospital of Zhengzhou University, Henan Provincial People’s Hospital, Henan Eye Institute, Henan Eye Institute, 7# Weiwu Road, Zhengzhou, 450003 Henan China

**Keywords:** Ephrin-A1, VEGF_165_, Plasma, Diabetic retinopathy, ELISA

## Abstract

**Background:**

To determine plasma ephrin-A1 and VEGF_165_ levels in a cohort of diabetic retinopathy patients.

**Methods:**

Plasma ephrin-A1 and VEGF_165_ levels in fifty-five subjects including 19 individuals without diabetes (non-DM), 16 patients with diabetes (DM) but without diabetic retinopathy, and 20 patients with diabetic retinopathy (DR), were determined by ELISA. Serum creatinine, total cholesterol, fasting blood glucose and HbA1c were also measured. One-way ANOVA, Kruskal-Wallis Test, Mann-Whitney U Test corrected by Bonferroni, Pearson Correlation Analysis and Spearman Correlation Coefficient Analysis were used for data analysis.

**Results:**

Ephrin-A1 expression could be detected in human plasma with an average of 1.52 ± 0.43 (mean ± SEM) ng/ml. In DR subjects, the plasma ephrin-A1concentration was 3.63 ± 4.63 ng/ml, which was significantly higher than that of the other two groups (non-DM: 0.27 ± 0.13 ng/ml, DM: 0.35 ± 0.34 ng/ml). The expression of VEGF_165_ in human plasma was 34.00 ± 42.55 pg/ml, with no statistical difference among the three groups. There was no correlation between ephrin-A1 and VEGF_165_ in human plasma, but there was a correlation between plasma ephrin-A1 and duration of diabetes.

**Conclusions:**

Plasma ephrin-A1 was highly expressed in patients with diabetic retinopathy, and there was no difference of plasma VEGF_165_ expression in patients with diabetic retinopathy compared to the other two groups, suggesting that changes of plasma ephrin-A1 may be a more sensitive biomarker than plasma VEGF_165_ in detecting diabetic retinopathy.

## Background

It is estimated that there are 382 million diabetic patients worldwide, and about one third of these patients have diabetic retinopathy (DR), part of them have diabetic macular edema. DR causes visual impairment in about 37 million people around the world [1]. About 60% of type 2 diabetes patients present DR of varying degrees 20 years after being diagnosed with diabetes. DR is induced by hyperglycemia, which leads to retinal vascular endothelial injury, retinal vascular loss, ischemia, and changes of leukocyte adhesion [2, 3]. Consequently, production of precursor substances of various angiogenic factors and inflammatory cytokines increased which induced abnormal neovascularization [4] and microvascular dysfunction.

In the pathogenesis process of DR, endothelial-derived growth factor (VEGF) is a major cytokine that promotes neovascularization, which leads the destruction of blood retinal barrier and indirectly promoting the progress of DR. Several spliced isoforms of VEGF present in human body with 121, 145, 165, 183,189, and 206 amino acids in length [5]. VEGF_165_ appears to be the most abundant and potent isoform in inducing neovascularization, followed by VEGF_121_ and VEGF_189_ [5, 6]. In vivo and in vitro experiments have shown that ischemia and hypoxia induces VEGF expression, which causes retinal neovascularization [7]. In addition, it has been shown that VEGF levels in the vitreous of DR patients were higher than those in the control group [8]. Intravitreal injection of anti-VEGF drugs has a certain therapeutic effect on PDR patients [9, 10]. However, there are disadvantages that anti-VEGF drugs require multiple intravitreal injections for the treatment of DR, that maybe causing preretinal tissue shrinkage and retinal detachment [11]. In addition, anti-VEGF drugs have adverse effects such a mild cerebrovascular accident and myocardial infarction. Therefore, it is necessary to find more appropriate approaches or new therapeutic targets.

Ephrins and the Eph receptors have been identified as key regulators of angiogenesis [12]. Class A Eph receptors have been shown to regulate postnatal angiogenesis in adults. Earlier studies have shown that ephrin-A1 stimulates the migration of cultured endothelial cells and induces corneal angiogenesis [13]. Expression of ephrin-A1 was found in the process of embryonic vascular development [12] and tumor growth [14]. These evidences indicate that ephrin-A1 plays an important role in angiogenesis. We became interested whether ephrin-A1 is expressed in human plasma, and whether the plasma ephrin-A1 expression was altered in diabetic retinopathy patients.

## Methods

### Subjects

This study was approved by institutional ethics committee in the Fenghua Hospital of Traditional Chinese Medicine. Informed consents in written format to publish these date were collected from each patient, who agreed and signed the consent to participate statement. Primary angle-closure suspect was inclusion criteria, because there were no reports about the association between ephrin-A1 and primary angle-closure suspect, or VEGF and primary angle-closure suspect in the literature. In addition, there is no vascular changes or cytokine changes that are associated with angiogenesis in primary angle-closure suspect. The recruited patients were enrolled between August 1, 2018 and October 31, 2019 with the following inclusion criteria:

1. Over 40 years of age.
2. Clinical diagnosis of primary angle-closure suspect, diabetes mellitus and DR.
3. Patients agreed to provide informed consent.

The definition of primary angle-closure suspect was in accordance with the International Society of Geographical and Epidemiologic Ophthalmology criteria [15]. Diabetes mellitus was diagnosed according to the international standards [16]. Diagnosis of DR was made based on an international standard [17].

The subjects with any diseases that presented neovascularization such as kidney disease and cancer, or any of those subjects received anti-VEGF therapy or operations were excluded.

A total of fifty-five patients from Fenghua District were recruited. Nineteen patients with primary angle-closure but without diabetes were enrolled in the non-DM group. Sixteen patients with primary angle-closure and diabetes were enrolled in DM group. Twenty patients with DR were enrolled in DR group, about 35% of patients with proliferative diabetic retinopathy.

After routine physical and eye examinations, electrocardiogram, clinical laboratory tests including liver and kidney function tests, routine ophthalmic examinations, fundus photograph and fluorescein angiography were performed.

The averaged duration time of type 2 DM in the diabetic group was 46 ± 18.5 months. The averaged duration time of type 2 DM with DR was 118 ± 49 months.

### Collection of blood samples

The blood samples were collected using ethylenediaminetetraacetic acid (EDTA) as an anticoagulant from the peripheral vein of each patient and transferred to Department of Clinical Laboratory, which is a section of Fenghua Hospital of Traditional Chinese Medicine. Samples were centrifuged immediately for 15 min at 1000 g. Then, plasma was collected and stored at − 80 °C before enzyme-linked immunosorbent assay (ELISA) measurement.

All individuals were informed of the purpose of the study and their informed consent was obtained. This study followed the tenets of the Declaration of Helsinki and was approved by the ethics committee of the institutional Fenghua Hospital of Traditional Chinese Medicine.

### Measurement of plasma Ephrin-A1 and VEGF_165_

Ephrin-A1 was measured using a commercial enzyme-linked immunosorbent assay (ELISA) kit (LSBio, Seattle, WA, USA) and VEGF_165_ was measured using a commercial enzyme-linked immunosorbent assay (ELISA) kit (R&D systems, Minneapolis, MN, USA), following the instructions. Results were obtained by a multifunction microplate reader (Molecular Devices Inc., Sunnyvale, CA, USA). In addition, 6 ml venous blood of subjects was taken for measurement of fasting blood glucose, HbA1c, serum creatinine, and total cholesterol levels.

### Statistical analysis

Statistical analyses were performed using SPSS 18.0 (SPSS Inc. Chicago, IL, USA). The data were shown as mean ± SD. After inspecting the distribution of the data, we assessed statistical significances with One-way ANOVA, Kruskal-Wallis Test, Mann-Whitney U Test corrected by Bonferroni. Correlations between any two of parameters including age, BMI, plasma ephrin-A1, VEGF_165_, fasting blood glucose levels, HbA1c, serum creatinine and total cholesterol were examined by Spearman’s Correlation Analysis. Correlation between ephrin-A1 and duration of diabetes was performed by Pearson Correlation Analysis. *P* < 0.05 was considered statistically significant.

## Results

Clinical and laboratory data were shown in Table 1. There was no statistical difference in age, sex, VEGF_165_, serum creatinine and total cholesterol among the three groups of subjects. No relationship between Ephrin-A1 and VEGF_165_ in human plasma had been found. There were differences in fasting blood glucose and HbA1c among the three groups. Compared to the non-DM subjects, DM patients and DR patients had higher fasting blood glucose and HbA1c (*P* < 0.05).

**Table 1:** Clinical and laboratory features of DR, DM and non-DM subjects

	non-DM (***n*** = 19)	DM (***n*** = 16)	DR (***n*** = 20)	***P***	***P Value 1***	***P Value 2***	***P Value 3***
Age (years)	60.53 ± 4.04	63.31 ± 4.97	60.25 ± 6.72	0.048	0.093	0.159	0.741
male/female	11/8	8/8	11/9				
BMI (kg/m2)	22.01 ± 2.25	24.48 ± 3.17	24.32 ± 3.66	0.032	0.068	1	0.072
FPG (mmol/l)	5.57 ± 0.62	8.91 ± 3.04	8.16 ± 2.57	<0.001	<0.001	> 0.05	<0.001
HbA1c(%)	5.33 ± 0.32	8.65 ± 2.56	8.22 ± 1.84	<0.001	<0.001	> 0.05	<0.001
VEGF165(pg/ml)	34.79 ± 38.65	24.43 ± 17.33	40.91 ± 58.01	0.093			
Ephrin-A1(ng/ml)	0.27 ± 0.13	0.35 ± 0.34	3.63 ± 4.63	<0.001	> 0.05	<0.001	<0.001
Serum creatinine (μg/ml)	54.32 ± 12.74	68.88 ± 34.04	70.10 ± 26.63	0.063			
Total cholesterol (mmol/l)	5.19 ± 1.09	5.41 ± 1.45	5.00 ± 1.00	0.064			
Duration of DM (months)	0	46 ± 18.5	118 ± 49				

*P* indicates *P* value of the three group; *P Value 1* indicates *P*_*adj*_ value of the non-DM group vs the DM group; *P Value 2* indicates *P*_*adj*_ value of the DM group vs the DR group; *P Value 3* indicates *P*_*adj*_ value of the non-DM group vs the DR group.

BMI between the three groups was analyzed using one-way ANOVA. Age, HbA1c, FPG, serum creatinine, total cholesterol, ephrin-A1 and VEGF were analyzed by Kruskal-Wallis Test.

The *P* values of serum creatinine, total cholesterol and VEGF were >0.05, suggesting that there was no statistical significance between the three groups. We found that the *P* values of age, HbA1c, fasting blood glucose and ephrin-A1 were >0.05, then Mann-Whitney U Test corrected by Bonferroni was further used for comparison between any two groups.

*FPG* Fasting plasma glucose. Values are means ± SD

**Fig. 1:**
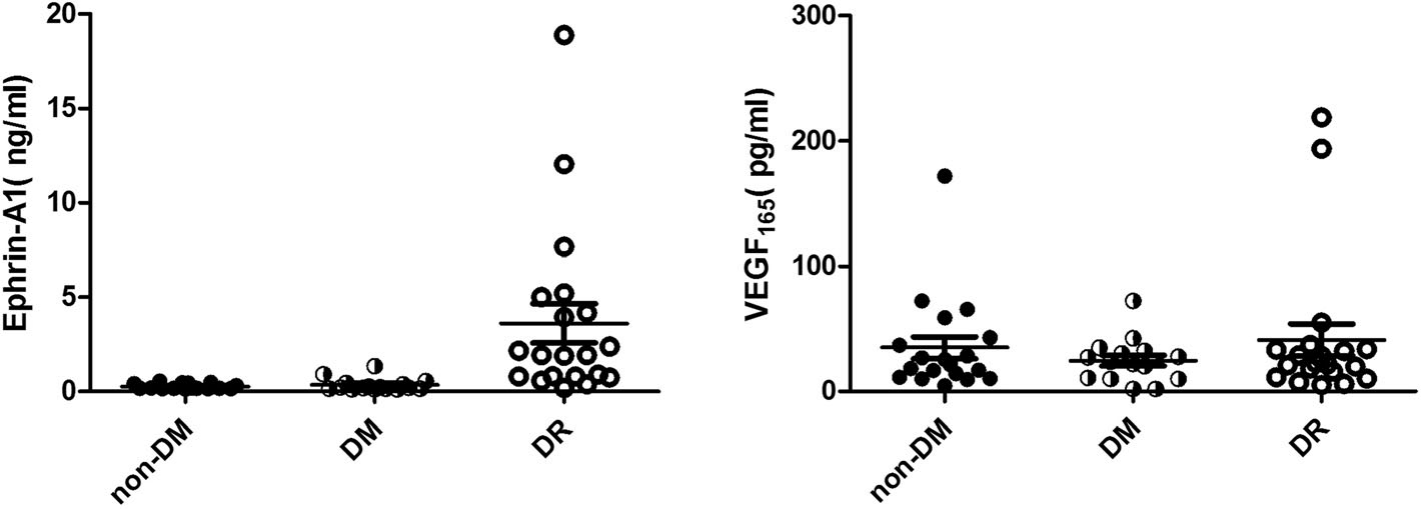
Plasma VEGF_165_ and Ephrin-A1 concentrations in DR and non-DR subjects. The concentration of ephrin-A1 of the DR subjects was significantly higher than that of the other two groups (*P* < 0.05). There was no statistical difference in VEGF_165_ among the three groups

Ephrin-A1 could be measured in human plasma, the averaged concentration of ephrin-A1 was 1.52 ± 0.43 (mean ± SEM) ng/ml. The concentration of ephrin-A1 in the DR subjects was significantly higher than that of the other two groups (*P* < 0.05)(Fig. 1). This difference of the ephrin-A1 concentration was not found between the non-DM subjects and the DM subjects.

Plasma ephrin-A1 was correlated with the duration of diabetes (*r* = 0.513,*P* < 0.05) and with Serum creatinine (*r* = 0.338,*P* < 0.05), but was not correlated with age, BMI, serum VEGF_165_, total cholesterol, fasting blood glucose and HbA1c in the DR patients, the DM subjects, and the non-DM subjects or all subjects.

## Discussion

Interaction between EphA receptor tyrosine kinases (RTKs) and ephrinA ligands is necessary for inducing of maximal neovascularization by VEGF [18]. EphA2 RTK is activated by VEGF through induction of ephrinA1 ligand [18]. Thus, we speculated that ephrin-A1 may be involved in the pathogenesis of DR and the ephrin-A1concentrations in DR patients may be changed. To the best of our knowledge, no such research has been conducted before. Meanwhile, there were no reports about the association between ephrin-A1 and primary angle-closure suspects, or VEGF and primary angle-closure suspects in the literature.

In this study, ephrin-A1 concentrations in the plasma of non-diabetic, diabetic and DR patients were measured in a cohort of subjects. The averaged ephrin-A1 concentration in human plasma was 1.52 ± 0.43 (mean ± SEM) ng/ml, indicating ephrin-A1 was expressed in human plasma. We found ephrin-A1 concentration in the DR group was higher than that in the non-diabetic and the diabetic groups, suggesting ephrin-A1 may be involved in the development of DR. There is evidence showed that ephrin-A1 inhibits VEGF-induced intracellular signaling and suppresses retinal neovascularization and blood-retinal barrier breakdown [19]. Previous studies reported that ephrin-A1 has also been shown to be expressed in the developing vasculature during embryogenesis [14] and in nenvascular cells during tumor growth [12]. However, where does the increased ephrin-A1 come from in diabetic retinopathy remains unknown, it may require further studies including multiple systems and organs.

We also measured the plasma concentrations of VEGF_165_ in these patients. We did not find difference in plasma VEGF_165_ among the three groups. This result was inconsistent with a previous report that the plasma VEGF_165_ concentration elevated in DR patients compared to non-DR controls [20]. Further studies are needed to confirm the changes of VEGF_165_ in the systemic circulation in DR patients.

## Conclusions

This study is the first report that ephrin-A1 is presented in circulation. Furthermore, ephrin-A1 expression is elevated in DR patients, suggesting it may be involved in the pathogenesis of DR. Whether it may become a biomarker for the disease deserves further studies. Where does the increase ephrin-A1 come from and the mechanism of ephrin-A1 upregulation requires further research.

## Data Availability

The datasets used and/or analyzed during the current study are available from the corresponding author on reasonable request.

## Abbreviations

VEGF: Endothelial-derived growth factor
DM: Diabetes
DR: Diabetic retinopathy
ELISA: Enzyme-linked immunosorbent assay
EDTA: Ethylenediaminetetraacetic acid
